# To explore the prognostic efficacy and mechanism of ABCC5 clinical scoring model in hepatocellular carcinoma

**DOI:** 10.3389/fonc.2025.1519533

**Published:** 2025-08-11

**Authors:** Yu Deng, Ning Yang, Chengyu Huang, Meiting Long, Junming Wu, Ke Mo, Zijun Li

**Affiliations:** ^1^ Department of General Practice, Guangdong Provincial People’s Hospital, Guangdong Academy of Medical Sciences, Southern Medical University, Guangzhou, China; ^2^ Experimental Center of Baiqian Gene (BIOQGene), YuanDong International Academy of Life Sciences, Hong Kong, Hong Kong SAR, China; ^3^ Systems Biology Research Center, Biology Institute, Guangxi Academy of Sciences, Nanning, Guangxi, China

**Keywords:** ABCC5, HCC, immune microenvironment, multi-omics data, drug targets

## Abstract

**Objective:**

The study found that ATP-binding cassette subfamily C member 5 (ABCC5) is highly expressed in hepatocellular carcinoma (HCC). It aims to explore ABCC5 role and prognostic value in HCC and uses the DrugBank database to identify potential therapeutic drugs targeting ABCC5, assessing its potential as a biomarker and treatment target for HCC.

**Methods:**

RNA-seq and clinical data from TCGA-LIHC and GSE76427 were analyzed to identify ABCC5-associated differentially expressed genes and miRNAs. Weighted gene co-expression network analysis (WGCNA) revealed co-expression modules, and survival analysis assessed prognostic significance. Experimental validation included qRT-PCR, Western blot, migration assays, and drug response studies using the ABCC5 inhibitor zidovudine (ZDV).

**Result:**

ABCC5 was significantly overexpressed in HCC (*p* < 0.001) and correlated with poor overall (*p* = 0.008) and recurrence-free survival (*p* < 0.0001). WGCNA identified the MEturquoise module (enriched in cell cycle and p53 pathways) strongly linked to ABCC5 (r = 0.54). Immune infiltration analysis showed ABCC5 high-expression associated with Treg accumulation (immune suppression) and reduced mast cells. ZDV suppressed ABCC5 expression (~50%), activated p53 signaling (p53↑2.0-fold), and inhibited HCC migration and proliferation more effectively than the ABCC5-specific inhibitor MK-571. Somatic mutations (5% missense) and methylation (cg14480679, r = -0.43) further implicated ABCC5 in HCC progression. The ABCC5-based prognostic model, validated by calibration curves, independently predicted survival (*p* < 0.0001).

**Conclusion:**

This study constructed an ABCC5 clinical model and discovered that ABCC5 can serve as both a prognostic biomarker and therapeutic target for HCC. Multi-omics analysis and experimental validation confirmed that ABCC5 drives HCC progression by participating in immune microenvironment reprogramming, affecting cell cycle progression, and regulating the p53 signaling pathway. The research not only identified potential diagnostic markers and therapeutic targets, but the established prognostic model also provides new insights for investigating HCC pathogenesis and clinical translation.

## 1 Introduction

Liver cancer is the sixth commonest malignancies and the third leading cause of cancer mortality worldwide in 2022 (1). Hepatocellular carcinoma (HCC) accounts for 90% of primary liver cancers (2). The morbidity and mortality of HCC rank the fourth and second in all malignancies in China (3). According to the latest data from Cancer Statistics, 2024, over 900,000 people die from liver cancer globally each year, with the five-year relative survival rate for HCC being only 18% (4). This low survival rate highlights the urgent need for early diagnosis and effective treatment. Therefore, there is an increasing urgency to identify new prognostic biomarkers, particularly molecular markers capable of accurately predicting patient prognosis.

Due to the insidious onset and rapid progression of HCC, many patients are diagnosed at an advanced stage, missing the opportunity for curative treatments such as hepatic resection or liver transplantation. For advanced HCC patients, treatment options include local ablation, radiotherapy, chemotherapy and molecular targeted therapies (5). Despite significant efforts over the past few decades to identify novel diagnostic and therapeutic targets, the clinical outcomes for most HCC patients remain suboptimal. Current imaging techniques lack sensitivity for early-stage HCC, and biomarkers like alpha-fetoprotein (AFP) have limited utility in early screening (6, 7). While targeted therapies and immunotherapies show potential, patient responses are variable, and issues such as drug resistance and side effects continue to pose challenges (8). To address these limitations, the identification of novel biomarkers is urgently required to enhance early diagnostic precision and unveil new therapeutic targets for personalized treatment strategies.

Aberrant expression of ATP-binding cassette subfamily C member 5 (ABCC5), a member of the ABC transporter family, is linked to various cancers and plays a crucial role in regulating cancer cell function (9, 10). ABCC5 is a prognostic marker for tumor progression and drug resistance, with low expression in normal hepatocytes but strong induction upon cellular damage from exogenous or endogenous agents (11), ABCC5 is crucial for drug absorption, distribution, metabolism, and excretion. It uses ATP hydrolysis to actively transport drugs from cells to the extracellular space, impacting their bioavailability (12). Studies show that ABCC5 is closely associated with key cancer traits like drug resistance, metastasis, and proliferation (13). By modulating critical molecules in the tumor microenvironment (TME), ABCC5 helps cancer cells evade therapy and potentially enhances invasion and metastasis. It is highly expressed in various malignancies and plays a role in inducing ferroptosis (14), increasing drug resistance through Pemetrexed (MTA) accumulation (13), and influencing immune cell differentiation (15). Given its regulation by multiple pathways across cancers, ABCC5 is a promising therapeutic target, particularly for overcoming drug resistance and metastasis.

Previous studies have largely overlooked the co-expression networks influenced by ABCC5, focusing primarily on its direct impact on cancer cells. However, ABCC5’s effects extend beyond these actions, working with other genes to regulate key molecules in the TME and affecting cancer cell behavior. This study highlights the importance of co-expression networks in understanding complex diseases and how ABCC5 collaborates with other genes to modulate tumor progression. By exploring ABCC5-regulated co-expression networks in HCC, we aim to identify additional biomarkers that can inform personalized treatments. We identified ABCC5 as a potential biomarker due to its high expression and poor prognosis in clinical settings, confirming its prognostic value through survival analysis (10). This research further investigates ABCC5 co-expression patterns, functional pathways, immune infiltration, mutation types, and methylation in HCC, assessing its potential in diagnosis, monitoring, and as a target for anti-cancer therapies. Through this approach, we offer a new perspective on ABCC5 role in HCC, paving the way for novel clinical applications and therapeutic strategies.

## 2 Methods

### 2.1 Research data source

We obtained RNA sequencing data and clinical information for HCC from the TCGA-LIHC project within The Cancer Genome Atlas (TCGA) database (16), which includes 374 HCC samples, 50 adjacent non-cancerous tissue samples, somatic mutation data, and copy number variation (CNV) information. To enhance the quality and reproducibility of the study, we employed strict patient selection criteria. Specifically, we ensured that only HCC samples with complete clinical data, including survival outcomes, were included, while excluding those with missing or inconsistent clinicopathological information (**Supplementary Table S1**). This rigorous approach ensures that the dataset is robust and representative of the target population. The RNA sequencing data was then converted from count values to transcripts per kilobase million (TPM) format for subsequent analyses. For external validation, we utilized the GSE76427 dataset from the Gene Expression Omnibus (GEO). The data supporting this study are publicly available from the TCGA data portal (https://portal.gdc.cancer.gov/projects/TCGA-LIHC) and the GSE76427 dataset (https://www.ncbi.nlm.nih.gov/geo/query/acc.cgi?acc=GSE76427).

### 2.2 Expression of ABCC5 gene in HCC

After normalizing the TCGA-HCC data using the limma package (17), we further applied the DESeq2 (18) method to adjust for sequencing depth variation across samples, ensuring more accurate gene expression levels and eliminating technical biases. Following normalization, principal component analysis (PCA) (19) was performed to analyze the data and sample groups. HCC samples were treated as the primary group, with adjacent normal tissue samples as the control. PCA revealed the distribution of samples and highlighted the expression differences of the ABCC5 gene between the groups. Finally, the transcript levels of ABCC5 in HCC were visualized using violin plots based on normalized data, while significance of transcript levels was represented by **** (*p* < 0.001).

### 2.3 Exploring the potential of the ABCC5 gene as a prognostic biomarker for HCC

After determining the transcript levels of the ABCC5 gene in HCC, we will explore the potential of ABCC5 as a diagnostic marker for HCC and perform Receiver Operating Characteristic analyze (ROC) analysis based on the R language pROC software package (20), AUC > 0.9 indicates excellent diagnostic performance. We determined the optimal cutoff point for ABCC5 expression using the minimum p-value method and divided the patients into ABCC5 high-expression and low-expression groups. Subsequently, Kaplan-Meier survival analysis was performed to compare the survival differences between the two groups, further evaluating the potential role of ABCC5 in clinical prognosis (21). The prognosis of HCC patients was analyzed for recurrence-free survival (RFS) and overall survival (OS). The results of survival analysis were compared by log-rank test, and *p* < 0.05 was considered significant.

### 2.4 Differential expression analysis of genes and miRNA

High expression of ABCC5 is associated with poor prognosis of patients with HCC. Further exploration of ABCC5 and differential dysregulated genes in HCC was conducted, and differential gene expression of mRNA and miRNA was analyzed in HCC Control, limma package (17) of samples from high expression group and low expression group of ABCC5. The significance threshold was set at *p* < 0.05 to assess whether the between-group differences were statistically meaningful. A p-value less than 0.05 indicates that the observed differences are unlikely to be due to chance, thus considered significant, while a p-value greater than 0.05 suggests the differences could be due to random variation, making them non-significant. Additionally, a |logFC| > 1 (equivalent to a fold change greater than 2) was applied to screen for differentially expressed genes (DEGs) and miRNAs (DEmiRNAs), ensuring that only biologically relevant changes in expression were considered. Combining *p* < 0.05 with |logFC| > 1 ensures both statistical significance and biological relevance, allowing for the identification of genuinely differentially expressed genes and miRNAs (17). The expression patterns of DEGs and DEmiRNAs between the Control-HCC and ABCC5 high- and low-expression groups were then further analyzed. In addition, the intersection of DEGs and DEmiRNAs in the group was obtained respectively, and DEGs that were uniformly up-regulated or down-regulated were identified as dysregulated genes of HCC in ABCC5.

### 2.5 Weighted Gene Co-expression Network Analysis

Weighted Gene Co-expression Network Analysis (WGCNA) was employed to analyze dysregulated genes associated with ABCC5 in HCC, aiming to identify co-expressed gene modules and explore genes significantly linked to ABCC5. It constructs gene co-expression networks, which help identify modules that are closely linked to clinical traits, such as HCC prognosis. In this study, dysregulated genes were processed using the WGCNA package (22) in R. Outlier samples were excluded to enhance data quality, and a weighted network was constructed to ensure gene similarity reflected their logarithmic connectivity. Following module detection and selection, hierarchical clustering with the hclust function (23) confirmed module structures and identified outliers. Branches of the clustering tree corresponded to distinct gene modules. Heatmaps of module-phenotype correlations were generated to identify modules significantly linked to clinical traits (*p* < 0.05, r > 0.5), with strongly correlated modules selected as candidates for further investigation of ABCC5-related pathways in HCC.

### 2.6 Functional enrichment and gene enrichment analysis

By exploring the biology of the above dysregulated genes, these dysregulated genes were investigated using the cluster Profiler software package (24), the Genetic Ontology (enrich GO), and the Kyoto Encyclopedia of Genes and Genomes (enrich KEGG Channel Enrichment Analysis), and the enrichment results were visualized through bubble plots(Bubble size represents the number of genes with -log10FDR > 1.5 for significant enrichment). Where the analysis used “c5.bp.v7.0.entrez” and “c2.cp.kegg.v7.0.symbols.gmt” as internal reference genomes. Then phenotypes and whole gene expression profiles were entered into Gene Set Enrichment Analysis (GSEA) (25), and finally the Molecular Signature Database (MsigDB) analyzed the background data (26) (http://www.broadinstitute.org/msigdb) to verify the signaling pathways that were significantly activated or repressed by these genes. Subsequently, GSEA results showed differential expression of genes activated/inhibited in the pathways, and the enrichment results were considered significant at *p* < 0.05.

### 2.7 Construction and validation of ABCC5-based clinical model

The biological functions of key modular genes were examined, and prognostically significant genes were identified. Univariate and multivariate Cox regression analyses were then conducted to establish a set of ABCC5-associated prognostic scoring genes. The correlation between the ABCC5-based score and clinical indicators (OS, RFS, stage, grade, and gender) was visualized (significant at *p* < 0.05), with transcription levels of these genes presented in a box plot. To evaluate the ABCC5-based score as an independent prognostic factor, Cox regression results were summarized in a forest plot (SingleCoxCutOff: 0.01; MulCoxCutOff: 0.01; HRcutoff: -1). Only factors significant in both univariate and multivariate analyses were considered independent prognostic indicators. The ABCC5-based clinical model was visualized with a histogram for clarity, and its prognostic value was further assessed through Kaplan-Meier and calibration curves (significant at *p* < 0.05). Gene ontology semantic similarity analysis was conducted using the GOSemSim package (27). Additionally, Decision Curve Analysis (DCA) (28) was employed to evaluate the clinical utility of the predictive model by quantifying its net benefit across various decision thresholds, providing a comprehensive assessment of its real-world applicability beyond traditional metrics like accuracy or AUC.

### 2.8 Relationship between ABCC5 gene expression and immune infiltration in HCC

The R-packet deconvolution algorithm CIBERSORT (29) was used to evaluate the correlation between ABCC5 gene expression and immune cell infiltration in HCC. Correlation heat map (30) shows the infiltration abundance of immune cells in the Control, ABCC5-High, and ABCC5-Low groups. To better understand whether ABCC5 promotes or inhibits the invasion of specific immune cells, correlation scatter plots were used to illustrate the relationship between ABCC5 expression and immune cell infiltration abundance (*p* < 0.05 is significant, r < 0 is negative correlation, r > 0 is positive correlation). Additionally, correlation scatter plots were employed to analyze the association between the ABCC5-based prognostic score, ABCC5-related genes, and the abundance of immune cell infiltration, immune checkpoint-related genes, and markers of tertiary lymphoid structures. The results were then visualized for clarity.

### 2.9 Exploration of upstream regulatory factors of ABCC5-based scoring genes

The lncRNA-KEGG network, transcription factors (TFs)-KEGG network, and RNA-binding proteins (RBPs) were constructed based on the RNA-Inter database and the TRRUST v2 database and Hypergeometric test method (31) by Pivot analysis (32), and also the results were visualizing the regulatory role of upstream regulatory factors on the ABCC5-based scoring gene set. Potential drug targets for the ABCC5-based scoring gene set were identified using the DrugBank database (https://www.drugbank.com) (33). The gene set was mapped to drug targets listed in DrugBank, focusing on drugs with evidence of action on genes within the ABCC5 module. Cytoscape software (34) was then used to visualize the interactions between ABCC5, its associated genes, and potential drug targets. This approach enables the identification of direct interactions between target genes and drug compounds, offering insights into the broader regulatory network involving ABCC5. In addition, the PDB database (http://www.rcsb.org/) (35) was used in this study to retrieve and download gene structural domains in PDB format and the PubChem database (http://pubchem.ncbi.nlm.nih.gov), which was a small molecule and RNAi reagent bioactivity data public repository, applied pyMOL software (36) and AutoDock software (37) for preprocessing and docking, and visualized the docking results.

### 2.10 Multi-omics landscape of global regulatory network of ABCC5-based scoring genes

This study also explored the multi-omics landscape of the global regulatory network of ABCC5 scoring gene, and used the R language maftools package (38) to explore the mutation situation and mutation details of ABCC5-based scoring gene. Further, the mutation details of ABCC5-based scoring gene were explored by using the CNV strip graph of ABCC5-based scoring gene from the GISTIC2.0 tool (39). In addition, the correlation between methylation modification level and transcription level was explored and the correlation scatter plot was drawn, and the correlation between ABCC5-based clinical score gene and methylation site were demonstrated by bubble map.

### 2.11 Cell lines and culture

The human hepatocellular carcinoma cell line HepG2 and breast cancer cell line MCF-7 were obtained from Procell Life Science & Technology Co., Ltd. (Wuhan, China). HepG2 cells were cultured in Minimum Essential Medium (MEM; Gibco, 31985070) supplemented with 10% fetal bovine serum (FBS; Hycezmbio, FBS500-H) and 1% penicillin-streptomycin (Hyclone, SV30010), whereas MCF-7 cells were maintained under identical conditions. All cells were cultured in a humidified incubator at 37°C with 5% CO_2_.

### 2.12 Cell revival and seeding

Frozen cells were rapidly thawed in a 37°C water bath, transferred into 15 mL centrifuge tubes containing 3 mL of complete medium, centrifuged at 1000 rpm for 5 minutes, and resuspended in 5 mL fresh medium. Cells were seeded in T25 flasks and incubated at 37°C with 5% CO_2_. Once cells reached 70–80% confluency, they were trypsinized, resuspended, and seeded into 6 cm dishes at 3×10^5^ cells/mL for subsequent treatment. Drug treatments were initiated when cells reached 70–80% confluency: 2 μL of DMSO, 30 mM MK-571 (final 30 μM), or 10 mM zidovudine (ZDV) (final 10 μM) were added to 2 mL of culture medium, and cells were incubated for 48 hours before assays.

### 2.13 Western blot analysis

To investigate the expression changes of the key transporter protein ABCC5 and regulatory factors MDM2 and P53 under experimental interventions, this study employed Western blotting technique. Specifically, treated cells were lysed using RIPA buffer (Beyotime, China) containing protease and phosphatase inhibitors. After protein concentration determination by BCA assay (Beyotime), equal amounts of protein (20-30 μg) were separated by 10% SDS-PAGE gel electrophoresis (optimal for 20–80 kDa proteins) and subsequently transferred to PVDF membranes (Millipore, USA). The membranes were blocked with 5% non-fat milk in TBST for 1 hour at room temperature, followed by overnight incubation at 4°C with primary antibodies against ABCC5 (1:1000, Abcam), MDM2 (1:1000, CST), P53 (1:1000, CST), and β-actin (1:3000, Proteintech) as loading control. After TBST washing, the membranes were incubated with HRP-conjugated secondary antibodies (1:5000, Proteintech) for 2 hours at room temperature. Protein bands were visualized using ECL chemiluminescent reagent (Thermo Fisher) and imaged with a ChemiDoc XRS+ system (Bio-Rad). Band intensity was quantified using Image Lab software by comparing the gray value ratios of target proteins to β-actin, enabling quantitative analysis of ABCC5-mediated drug efflux function and its regulatory relationship with the MDM2-P53 signaling pathway.

### 2.14 Quantitative real-time PCR

To quantify the expression levels of target genes under experimental conditions, qRT-PCR was performed following rigorous RNA isolation and reverse transcription protocols. Total RNA was isolated from tissues or cultured cells using SparKZol Reagent (SparkJade, China), with homogenization in TRIzol to ensure complete lysis, followed by phase separation via chloroform addition and RNA precipitation with isopropanol. The resulting RNA pellet was washed with 75% ethanol to remove contaminants, and purity was verified by NanoDrop spectrophotometry (A260/A280 ratio 1.7–2.1). For cDNA synthesis, 1 μg of total RNA was first treated with 8× gDNA remover (42°C, 2 min) to eliminate genomic DNA contamination, then reverse transcribed using 5× All-In-One RT SuperMix (ABclonal, China) under optimized conditions (37°C for 15 min, 85°C for 5 sec). qRT-PCR amplification was conducted with TB Green^®^ Premix Ex Taq™ II (Takara, Japan) on a LightCycler 480 system (Roche), using 10 μL reactions containing 5 μL master mix, 0.25 μL each of gene-specific primers (10 μM), 1 μL cDNA, and 3.5 μL RNase-free water. Thermal cycling included initial denaturation (95°C, 30 s), 40 cycles of amplification (95°C for 10 s, 60°C for 20 s, 72°C for 30 s), and melting curve analysis to confirm primer specificity. GAPDH served as the endogenous control, and relative gene expression was calculated using the 2^−ΔΔCt^ method to compare transcriptional changes across experimental groups.

### 2.15 Flow cytometry for apoptosis detection

Apoptosis was evaluated by Annexin V-APC/7-AAD double staining (KeyGEN BioTECH, China) to assess the effect of miR-203a-3p mimics and RSK4 on MCF-7 cell apoptosis, determining whether miR-203a-3p overexpression or RSK4 modulation influences cell survival. Treated HepG2 cells were collected, washed twice with cold PBS, and resuspended in 500 μL 1× binding buffer. Cells were incubated with 5 μL Annexin V-APC and 10 μL 7-AAD for 5 minutes at room temperature in the dark. Data acquisition was performed using a CytoFLEX flow cytometer (Beckman Coulter), and results were analyzed with FlowJo software (v10.0) to quantify the apoptotic rates across different treatment groups.

### 2.16 Cell cycle analysis

Cells were harvested after treatment, washed with PBS, and fixed in 70% cold ethanol overnight at 4°C to prepare for DNA content analysis, which would reveal whether miR-203a-3p mimics or RSK4 overexpression alters cell cycle progression in MCF-7 cells. After washing, cells were stained with 50 μg/mL propidium iodide (PI) and 50 μg/mL RNase A at 37°C for 30 minutes in the dark to measure DNA content distribution and determine the proportion of cells in G0/G1, S, and G2/M phases. Cell cycle distribution was analyzed on a CytoFLEX flow cytometer, and data were processed with ModFit LT software to compare cell cycle profiles among the experimental groups and assess potential cell cycle arrest or dysregulation induced by miR-203a-3p or RSK4.

### 2.17 Wound healing assay

To evaluate the effects of ABCC5 inhibition and ZDV treatment on cell migration, MCF-7 cells were seeded into 6-well plates and grown to confluency. A sterile 200 μL pipette tip was used to create a linear scratch across the monolayer. Cells were washed three times with PBS to remove debris and incubated in serum-free medium containing DMSO (solvent control), MK-571 (30 μM, an ABCC5-specific inhibitor as a positive control), or ZDV (10 μM, experimental group). Images were taken at 0 h and 48 h using an inverted microscope (MF53, Mingmei) at 4× magnification. The wound area was measured using ImageJ software, and the percentage of wound closure was calculated.

### 2.18 Data analysis and statistics

All the biological information analysis in this study were conducted based on Bioinforcloud platform (http://www.bioinforcloud.org.cn). The t-test, one-way ANOVA, and Wilcoxon rank-sum test were used to assess data distribution normality and homogeneity of variance in each group. Variables with a normal distribution are presented as mean ± standard deviation, while variables without a normal distribution are presented as median (quartiles). A p.value < 0.05 was considered statistically significant.

## 3 Results

### 3.1 Expression and prognosis of ABCC5 in HCC

**Figure 1:**
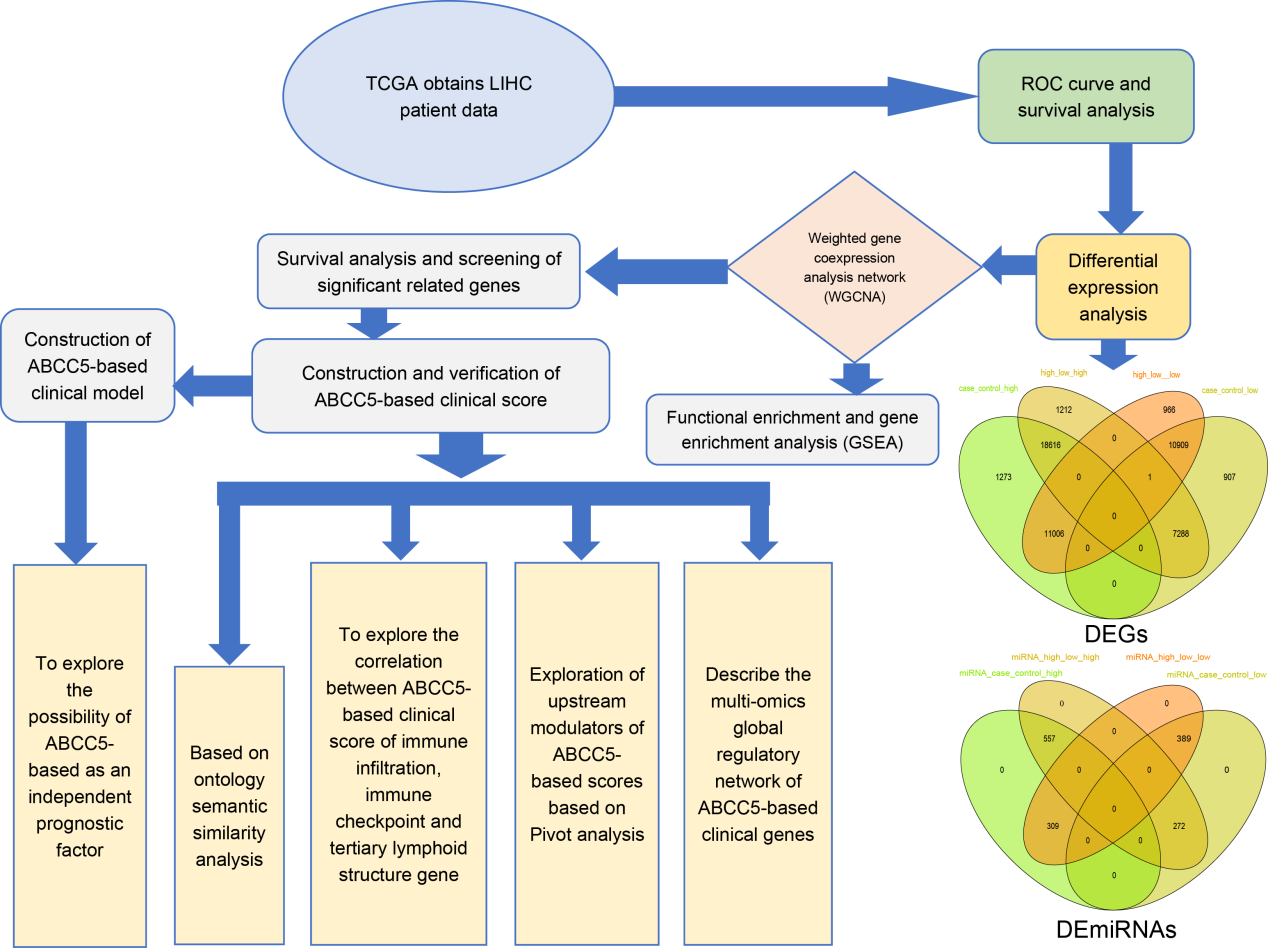
Workflow diagram. This study was mainly divided into five parts. In the first part, we used the TCGA database to download data related to HCC and performed ROC curve analysis and survival analysis. In the second part, we identified the differentially expressed genes of HCC by differential expression analysis. In the third part, we screened out dysregulated genes significantly related to ABCC5 expression in HCC for WGCNA and enrichment analysis. Then, we screened out the prognostic genes and target genes with significant prognosis and used univariate and multivariate COX regression analysis to construct an ABCC5-based Cox score based on the prognostic genes, and target genes and explored the relationship between score and score-related genes and prognosis and clinical indicators. Then, we further constructed an ABCC5-based scoring clinical model on top of the score and clinical index structure, studied the relationship between model genes and immune microenvironment, upstream modulators and potential drug targets, and constructed a multi-omics global regulatory network.

**Figure 2:**
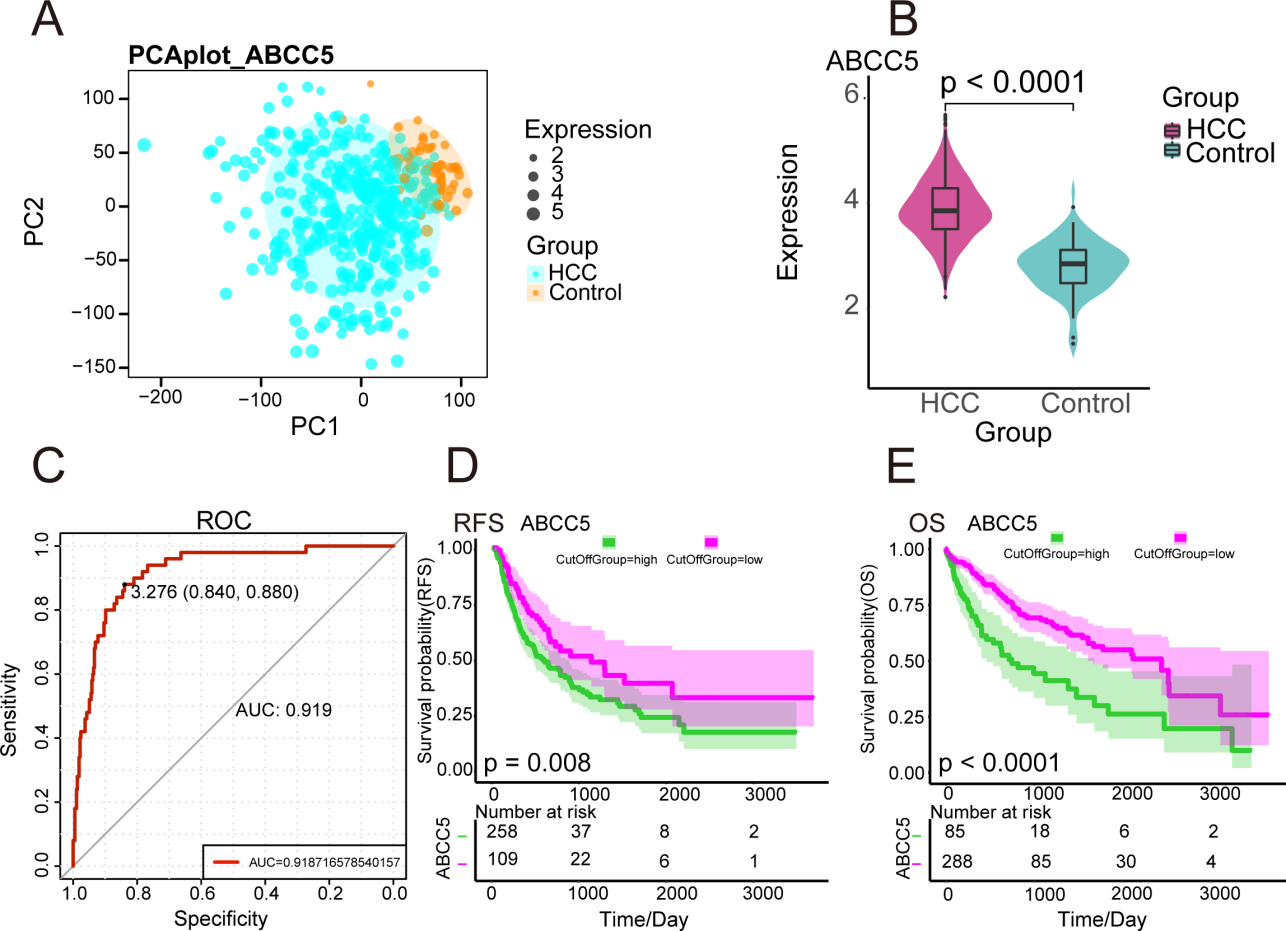
Expression and prognosis of ABCC5 in HCC. **(A)**. PCA density map mapping ABCC5 expression values; **(B)**. Violin diagram showing ABCC5 transcription levels in HCC. **** represents p-value < 0.0001; **(C)**. ROC diagnostic curve shows the diagnostic potential of ABCC5 for HCC; **(D)**. Survival curve showing the prognostic potential of ABCC5 for HCC OS; **(E)**. Survival curve showing the prognostic potential of RFS for HCC treated with ABCC5.

The workflow diagram of this study was shown in Figure (**Figure 1**). We analyzed the gene expression data of 424 HCC samples and 50 paracancerous tissue samples in the TGCA database. PCA revealed clear differentiation between HCC and control samples (**Figure 2A**), with high intra-group similarity and reproducibility. Notably, ABCC5 expression was significantly higher in HCC samples compared to controls (**Figure 2B**). In HCC, its elevated expression is linked to these processes, underlining its significant contribution to the disease’s biological mechanisms (15). Subsequently, we performed ROC curve analysis (**Figure 2C**), and the results showed that ABCC5 had good diagnostic potential for HCC (AUC = 0.9187). Immediately following, the expression of ABCC5 in HCC patients was explored, its effect on RFS and OS. The results of the specific analysis showed that the survival rate of the ABCC5 high-expression group was lower than that of the low-expression group in HCC patients, and ABCC5 overexpression was negatively correlated with OS (*p* = 0.008), and RFS (*p* < 0.0001) (**Figures 2D, E**). In conclusion, ABCC5 was significantly overexpressed in HCC patients, and survival analyses showed that poor prognosis in HCC patients was associated with ABCC5 overexpression. Therefore, it was a research direction to study the effect of ABCC5 expression process on poor prognosis of HCC patients. The ABCC5 offers significant prognostic value in HCC. Sensitivity and specificity analyses show high sensitivity in identifying high-risk patients and strong specificity in excluding low-risk individuals.

### 3.2 The gene co-expression module characterized the global regulatory pattern of ABCC5 in HCC

**Figure 3:**
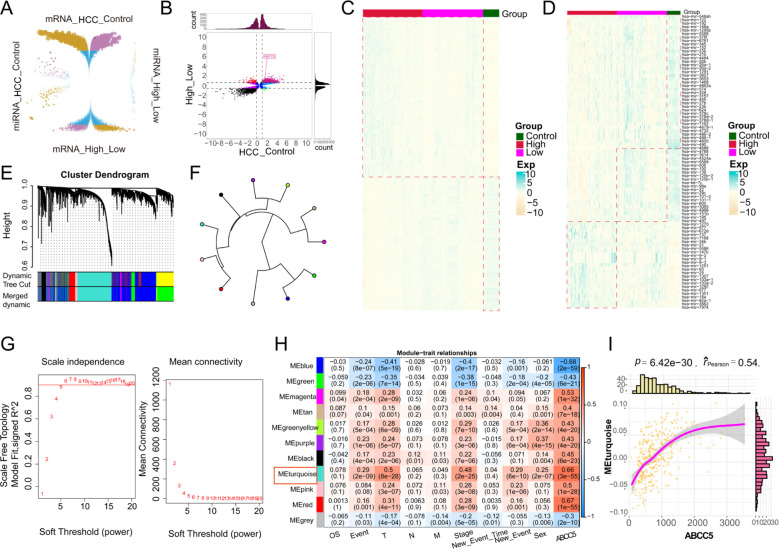
The gene co-expression module characterized the global regulatory pattern of ABCC5 in HCC. **(A)**. The wreath volcano map showed the DEGs and DEmiRNAs of ABCC5 in the control group and HCC, as well as in the high expression group and the low expression group; **(B)**. The potential role of differentially expressed genes and miRNAs affected by ABCC5 in HCC was demonstrated by the nine-grid scatter plot; **(C)**. The heat map showed the expression of DEmRNAs in the Control-ABCC5 high expression group and the low expression group; **(D)**. The heat map showed the expression of DEmiRNAs in the Control-ABCC5 high expression group and the low expression group; **(E)**. The module cluster tree shows the gene modules represented under different branches; **(F)**. Circular tree diagram of ABCC5 co-expression module adjacency; **(G)**. Display the selected digital threshold of the WGCNA soft threshold; **(H)**. Module correlation heat maps show the correlation between gene co-expression modules and ABCC5 and clinical features; **(I)**. Correlation scatter plots show the correlation between ABCC5 and co-expression modules.

To investigate the impact of high ABCC5 expression on HCC, we analyzed 19,746 DEGs and 485 DEmiRNAs that showed significant effects between HCC and control groups (**Figure 3A**). We then identified 8,606 DEGs and 152 DEmiRNAs that were differentially expressed between the ABCC5 high- and low-expression groups. Among these, 1,646 DEGs were consistently up- or down-regulated, including 714 up-regulated genes, 932 down-regulated genes, and 32 consistently altered DEmiRNAs. These genes were considered dysregulated in HCC and associated with high ABCC5 expression (**Figure 3B**). Heatmaps further illustrated the distribution of dysregulated genes and miRNAs in the control, ABCC5 high-expression, and ABCC5 low-expression groups (**Figures 3C, D**). Additionally, WGCNA analysis revealed that the gene co-expression networks followed the scale-free distribution and exhibited strong connectivity (**Figure 3E**). Eleven gene modules were identified, with the MEturquoise module showing a significant positive correlation with clinical features and HCC prognosis (**Figures 3F–H**). Correlation analysis confirmed a strong relationship between the MEturquoise module genes and ABCC5 (*p* < 0.001, r = 0.54) (**Figure 3I**). The ABCC5 scoring model, integrating expression levels with clinical data, aids in personalized treatment, prognostic risk assessment, and predicting tumor progression and chemotherapy response, ultimately guiding clinical strategies and improving prognosis.

### 3.3 ABCC5 gene in MEturquoise module is significantly involved in HCC disease-related biological functions and signaling pathways

**Figure 4:**
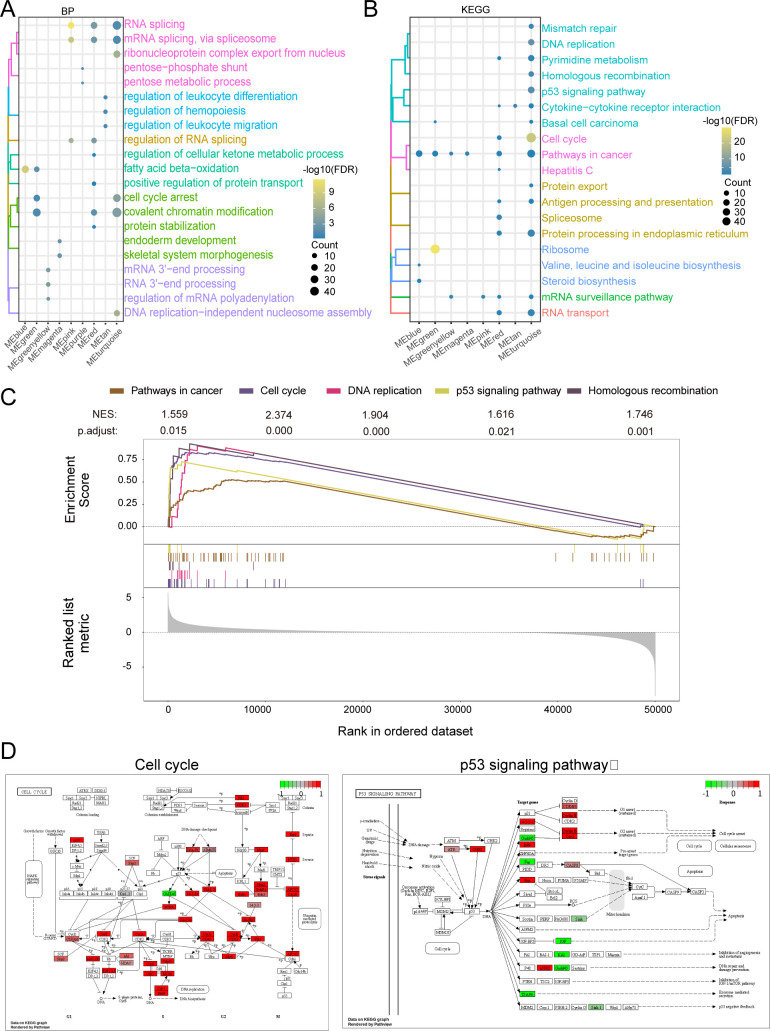
ABCC5 gene in MEturquoise module is significantly involved in HCC disease-related biological functions and signaling pathways. **(A)**. The cluster analysis bubble map showed the biological functions of the module genes significantly regulated with ABCC5; **(B)**. The bubble map of cluster analysis showed the signaling pathways significantly regulated by ABCC5 in the module genes most associated with ABCC5; **(C)**. The GSEA map showed the signaling pathway of significant enrichment of the module genes most associated with ABCC5; **(D)**. Pathway map shows the signaling pathways that were significantly activated/inhibited by ABCC5.

By functional enrichment of MEturquoise module genes. The genes in the module were found to be significantly involved in biological processes (BP) such as RNA splicing, covalent chromatin modification (**Figure 4A**) and significantly involved in KEGG signaling pathways such as pathways in the cell cycle and in cancer (**Figure 4B**). GSEA analysis revealed significant activation of genes in key pathways, such as Pathways in Cancer (NES = 1.559, P.adjust = 0.015), Cell Cycle (NES = 2.374, P.adjust < 0.001), DNA Replication (NES = 1.904, P.adjust < 0.001), p53 Signaling (NES = 1.616, P.adjust = 0.021), and Homologous Recombination (NES = 1.746, P.adjust = 0.001) (**Figure 4C**). These findings suggest that these pathways may play a crucial role in ABCC5-mediated HCC progression. Finally demonstrate that ABCC5 scoring gene expression changes mapped to specific metabolic networks such as cell cycle and P53 signaling pathways (**Figure 4D**).

Taken together, the findings suggest that the MEturquoise gene module is significantly involved in biological processes related to cell cycle, cell differentiation and apoptosis. Among them, the cell cycle can regulate the complex interactions between proteins, enzymes and cytokine signaling pathways, which are essential for cell proliferation, growth and repair. This suggests that the onset, progression and metastasis of HCC disease are closely related to the cell cycle. In this regard, using cell cycle regulation in combination with relevant inhibitory drugs can improve the sensitivity of chemotherapeutic drugs to tumor cells.

### 3.4 Exploring the prognostic diagnostic efficacy of ABCC5 in a clinical model of HCC

**Figure 5:**
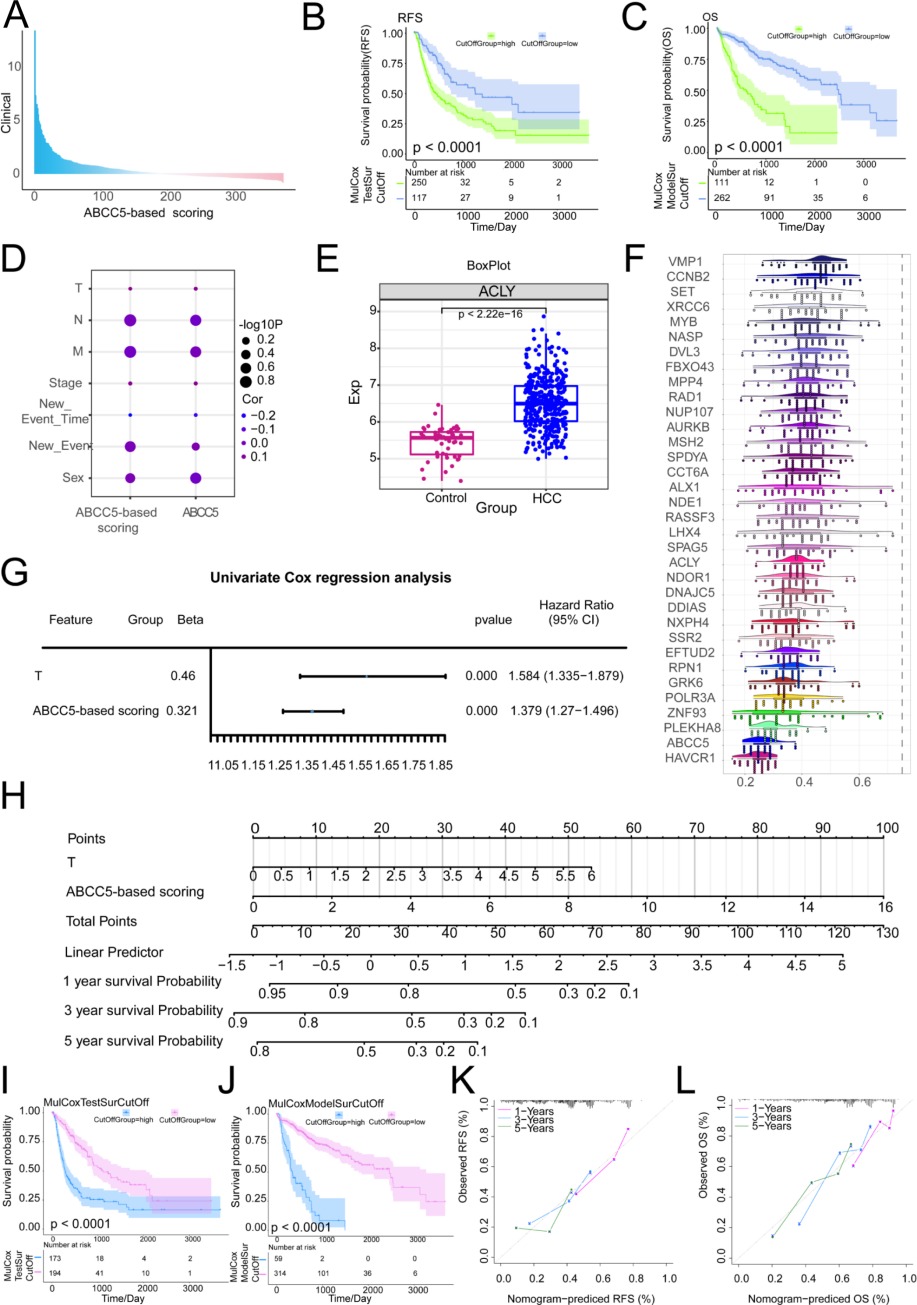
Exploring the prognostic diagnostic efficacy of ABCC5 in a clinical model of HCC. **(A)**. Bar chart showing multifactor Cox scores of gene sets associated with ABCC5 expression in clinical samples from HCC patients; **(B)**. Survival curve displays the RFS survival curve of the ABCC5-based score; **(C)**. Survival curve Displays the OS survival curve based on the ABCC5-based score; **(D)**. Bubble map showing the correlation between Cox prognostic score genes and clinical indicators; **(E)**. Box diagram shows the transcriptional expression level of ABCC5 in HCC; **(F)**. Cloud and rain maps show biological associations of scoring genes; **(G)**. Forest maps demonstrate the multifactorial prognostic efficacy of ABCC5-based scores and clinical indicators; **(H)**. Model histogram shows the ABCC5-based clinical model; **(I)**. Survival curves demonstrate the prognostic potential of the ABCC5-based scoring clinical model for RFS; **(J)**. Survival curves demonstrate the prognostic potential of OS in the ABCC5-based clinical model; **(K)**. Calibration curves demonstrate the RFS prognostic potential of the ABCC5-based scoring clinical model; **(L)**. Calibration curves demonstrate the prognostic potential of OS in the ABCC5-based clinical model.

The prognostic relevance of MEturquoise module genes to HCC was further explored. The MEturquoise module genes were subjected to survival analysis and subsequently screened for RFS (*p* < 0.0001) and OS (*p* < 0.0001) significance of the prognostic scoring genes and target genes (**Supplementary Table S2**). Construction and validation of ABCC5-based clinical scores (**Supplementary Table S3**). A Cox multifactorial prognostic score for the ABCC5-expressed functional genome was subsequently performed (**Figure 5A**). In the survival curves, we found that in OS (*p* < 0.0001), the ABCC5 high-expression group had higher overall survival time and survival risk than the HCC low-expression group (**Figure 5B**). In RFS (*p* < 0.0001), the mortality rate and risk of death were higher in the ABCC5 high-expression group compared to the low-expression group at all time points (**Figure 5C**). Bubble plots showed significant associations of the ABCC5 gene and ABCC5-based scores with clinical indicators (gender, stage, grading, among others) (**Figure 5D**). Several genes based on the ABCC5 score, such as ACLY (**Figure 5E**), exhibited significantly different transcript expression levels in HCC compared to controls. GO semantic similarity analysis further revealed that these genes were biologically related (**Figure 5F**). Subsequent explorations revealed that ABCC5-based scores and Tumor stage could serve as independent risk factors for HCC prognosis (**Figure 5G**). Combining these findings, this study also constructed a column-line graph prediction model containing ABCC5-based scores and Tumor staging, which predicted the survival of HCC patients at 1, 3, and 5 years (**Figure 5H**). The prognostic potential of the ABCC5 score-based clinical model in HCC was explored and validated (**Figures 5I, J**), showing significant survival prognostic potential. The calibration curves indicated that the column-line graph model accurately predicted 1, 3 and 5-year survival in HCC patients (**Figures 5K, L**). Additionally, we further explored whether incorporating tumor staging (Additional **Supplementary Figure S1**) could enhance the predictive power of the ABCC5 score model. The results indicated that the model with tumor staging did not outperform the model using ABCC5 score alone. Subsequently, we performed a simple validation of the model’s generalizability using a liver cancer patient dataset from the Gene Expression Omnibus database (Additional **Supplementary Figure S2**), and the results showed good generalizability. Based on these findings, further exploration of the genes in the MEturquoise module was conducted.

### 3.5 ABCC5-based scoring gene expression dysregulation reprograms the HCC immune microenvironment

**Figure 6:**
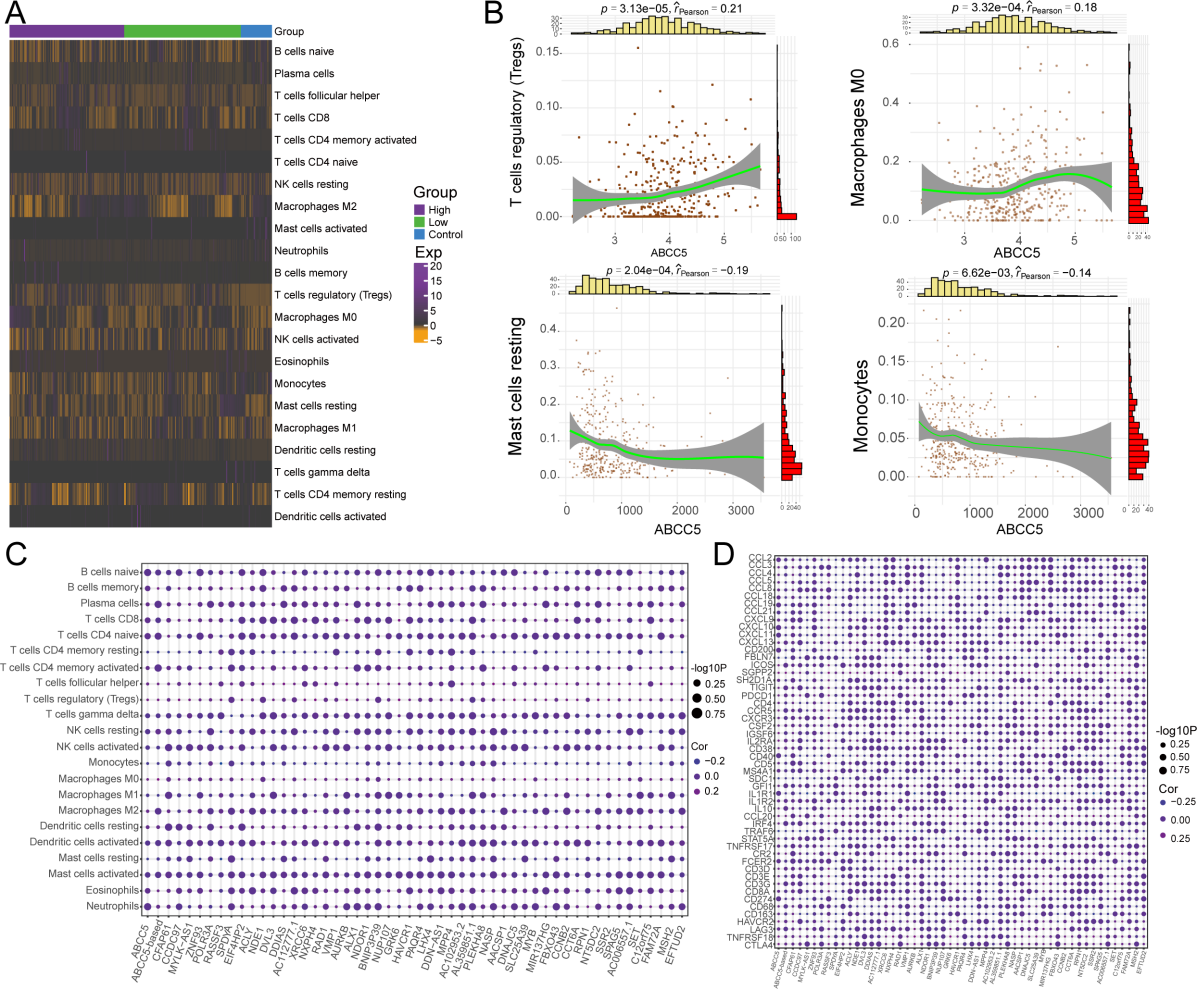
ABCC5-based scoring gene expression dysregulation reprograms the HCC immune microenvironment. **(A)**. Heat maps showed the infiltration abundance of Control-ABCC5 high expression group and low expression group; **(B)**. Scatter plots showed the correlation between the abundance of partial immune cell infiltration and ABCC5 expression; **(C)**. Bubble map shows the correlation between ABCC5-based scores and scoring genes and the abundance of immune cell infiltration; **(D)**. Bubble map showing the correlation between ABCC5-based scores and immune checkpoint related genes and tertiary lymphoid tissue marker scoring genes.

We evaluated immune cell control and infiltration levels in HCC, comparing ABCC5-high and ABCC5-low groups. The heatmap (**Figure 6A**) illustrates the immune cell proportions in HCC patients and controls. Correlation analysis revealed that high ABCC5 expression was positively correlated with Tregs and macrophages M0 infiltration, but negatively correlated with resting mast cells and monocytes (**Figure 6B**). This is consistent with studies linking Tregs to immune evasion and tumor progression in various cancers (40, 41). Specifically, ABCC5 correlation with Tregs suggests that ABCC5 may modulate the tumor immune microenvironment, facilitating immune escape by promoting Tregs accumulation, which suppresses anti-tumor immune responses (42). Furthermore, ABCC5-based scoring and gene analysis showed a significant correlation with the abundance of immune cells, immune checkpoint genes, and tertiary lymphoid structure markers (**Figures 6C, D**). This suggests that ABCC5 may influence responses to immunotherapy. Literature indicates that higher Treg levels are associated with reduced efficacy of immune checkpoint inhibitors (43, 44). Thus, ABCC5 may serve as both a key regulator of the immune microenvironment and a potential biomarker for predicting immunotherapy outcomes. These findings underscore ABCC5 role in immune evasion in HCC, offering insights for personalized immunotherapy strategies.

### 3.6 Upstream regulators of ABCC5-based clinical score gene set

**Figure 7:**
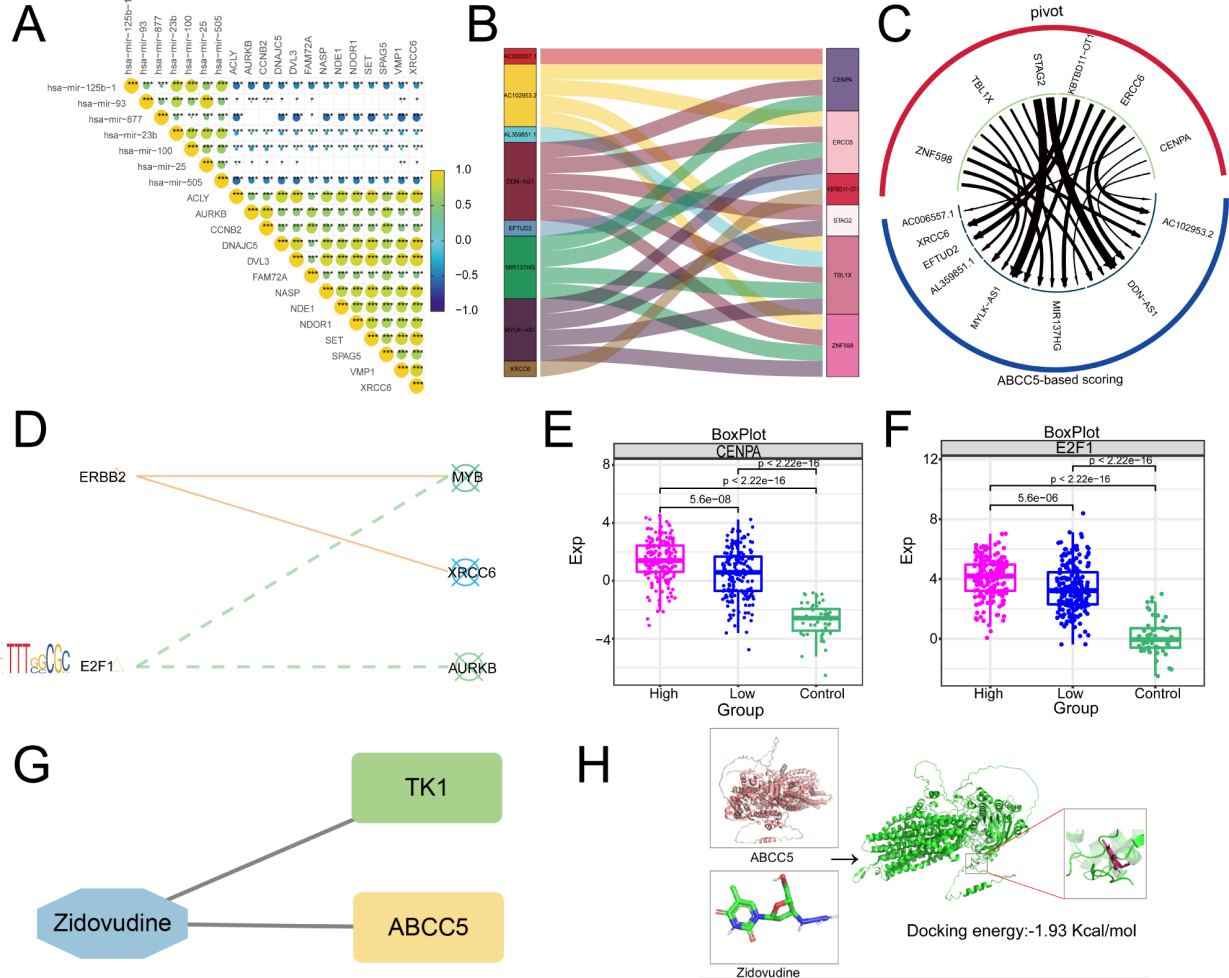
Upstream regulators based on ABCC5 score gene set. **(A)**. Triangle-correlated heat maps miRNAs reveal the regulatory effect of miRNAs on the gene set of clinical scores based on ABCC5; **(B)**. Sankey map showed the regulatory effect of lncRNA on the gene set of clinical scores based on ABCC5; **(C)**. Circular network diagram showing the regulatory effect of RBPs on the gene set of clinical scores based on ABCC5; **(D)**. The bubble line motif-Logo reveals the regulatory effect of TFs on the gene set of ABCC5 clinical score; **(E)**. The box diagram shows the transcriptional expression level of RBPs; **(F)**. The box diagram shows the transcription expression level of TFs; **(G)**. Network diagram showing potential drug targets based on the ABCC5 clinical score gene set; **(H)**. Docking diagram of a drug target molecule, the lower the binding energy (binding_energy: -1.93 Kcal/mol), the higher the docking potential energy. (Statistical significance notation: * represents p-value < 0.05; ** represents p-value < 0.01; *** represents p-value < 0.001; **** represents p-value < 0.0001.)

Then, based on Pivot analysis, upstream regulators of the clinical indicator gene set of ABCC5, including miRNAs, lncRNAs, RBPs, and TFs, were explored. Among them, there were 7 upstream miRNAs regulating the clinical indicator gene set of ABCC5, including has-mir-25b-1, among others (**Figure 7A**). Upstream lncRNAs include XRCC6, among others (**Figure 7B**). RBPs, including CENPA (**Figure 7C**), are key components in gene expression regulation. Additionally, transcription factors linked to the ABCC5-based process model were identified, highlighting the pathway’s complex regulatory network (**Figure 7D**). As visualized in the box line plot of the RBPs, the median of the High group in CENPA was the highest and the median of the Control group was the lowest (**Figure 7E**). Similarly, the box line plot of TFs has the highest median for the High-expression group and the lowest median for the Control group in E2F1 (**Figure 7F**). Subsequently, we also identified potential drug targets for the ABCC5-based scoring gene set, and the network diagram showed that TK1 and ABCC5 could be regulated by ZDV drug (**Figure 7G**). Immediately following the visualization of docking by molecular docking, the result was that the receptor ABCC5 and the ligand ZDV obtained 50 binding sites with targeted binding potential (**Figure 7H**). The results indicated that the ZDV drug has a good docking potential with ABCC5, suggesting a potential diagnostic site for HCC.

### 3.7 ZDV Exerts Synergistic Antitumor Effects by Activating the p53 Signaling Pathway Through Inhibition of ABCC5

**Figure 8:**
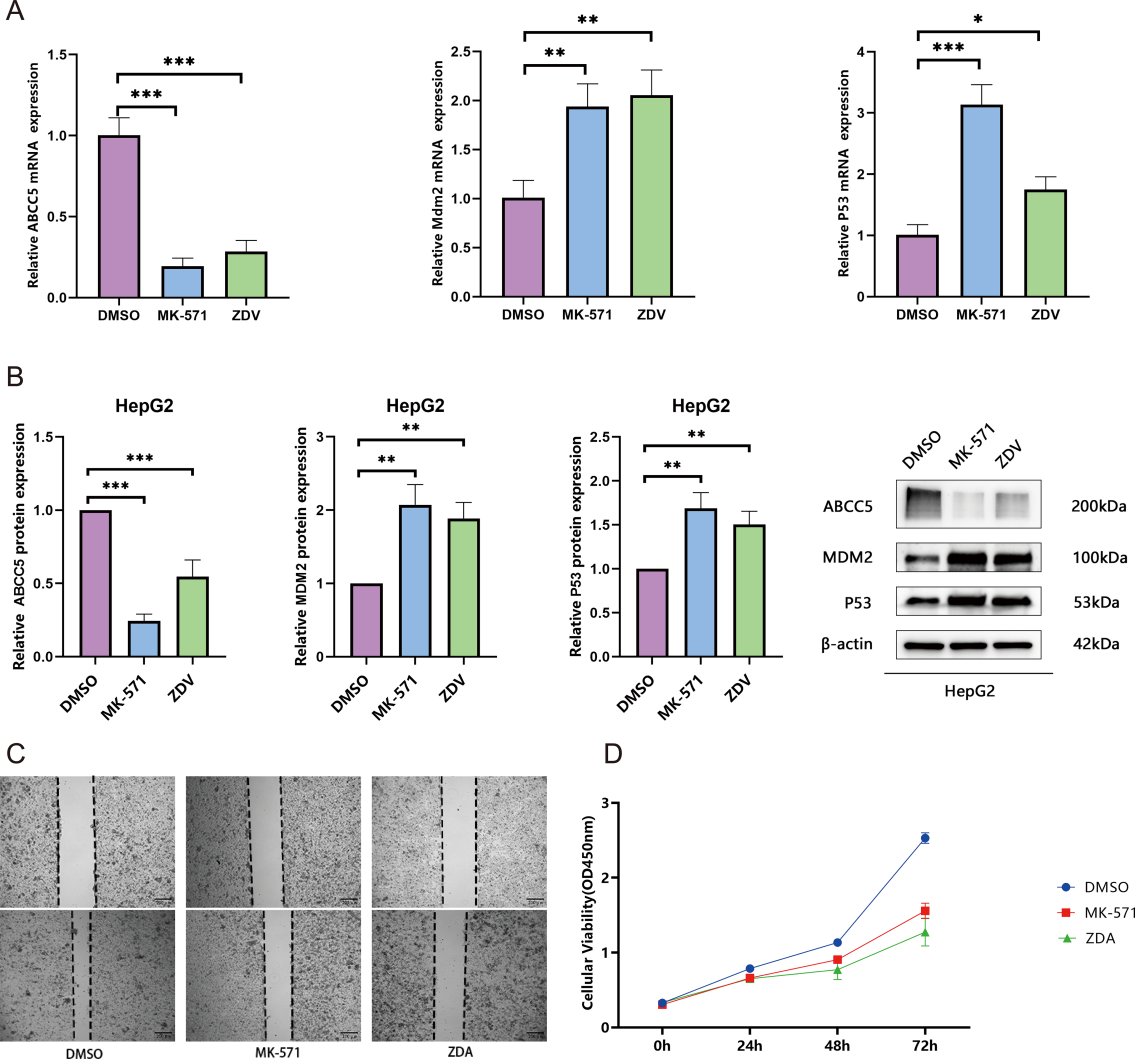
Zidovudine exerts synergistic antitumor effects by activating the p53 signaling pathway through inhibition of ABCC5. **(A)**. mRNA expression of ABCC5, Mdm2, and p53. qRT-PCR analysis revealed significant downregulation of ABCC5 and activation of the p53 signaling pathway (**p < 0.01, ***p < 0.001); **(B)**. Protein expression of ABCC5, MDM2, and p53. Western blot analysis confirmed suppression of ABCC5 protein and upregulation of p53 pathway-related proteins (**p < 0.01, ***p < 0.001); **(C)**. Wound-healing assay demonstrated that both MK-571 and ZDV treatment for 48 h significantly inhibited MCF-7 cell migration (**p < 0.01, ***p < 0.001); **(D)**. CCK-8 assay showed that, compared to the DMSO control (72 h OD=2.6), both ZDV (1.5) and MK-571 (1.9) significantly suppressed cell proliferation, with ZDV exhibiting a stronger inhibitory effect. Statistical significance notation: * represents p-value < 0.05; ** represents p-value < 0.01; *** represents p-value < 0.001; **** represent p-value < 0.0001.

This study systematically validated the anti- HCC mechanism of ZDV through multi-omics experiments. qRT-PCR results showed that ZDV significantly downregulated ABCC5 mRNA expression (decreased to 0.5-fold; *p* < 0.001) and activated the p53 pathway (Tp53 increased 2.0-fold, Mdm2 increased 2.1-fold; *p* < 0.01) (**Figure 8A**). Western blot analysis confirmed that ZDV reduced ABCC5 protein levels while upregulating p53 and MDM2 protein expression (**Figure 8B**). A cell scratch assay demonstrated that ZDV effectively inhibited HCC cell migration (**Figure 8C**). CCK-8 assays further revealed that ZDV exhibited stronger proliferation inhibition (72h OD 1.5) compared to the ABCC5-specific inhibitor MK-571 (1.9) (**Figure 8D**). In summary, ZDV activates the p53 signaling pathway by targeting and inhibiting ABCC5, while potentially exerting stronger anti-HCC effects through other synergistic mechanisms, providing a novel multi-target intervention strategy for HCC treatment.

### 3.8 Multi-omics landscape of global regulatory network of ABCC5-based clinical scoring genes

**Figure 9:**
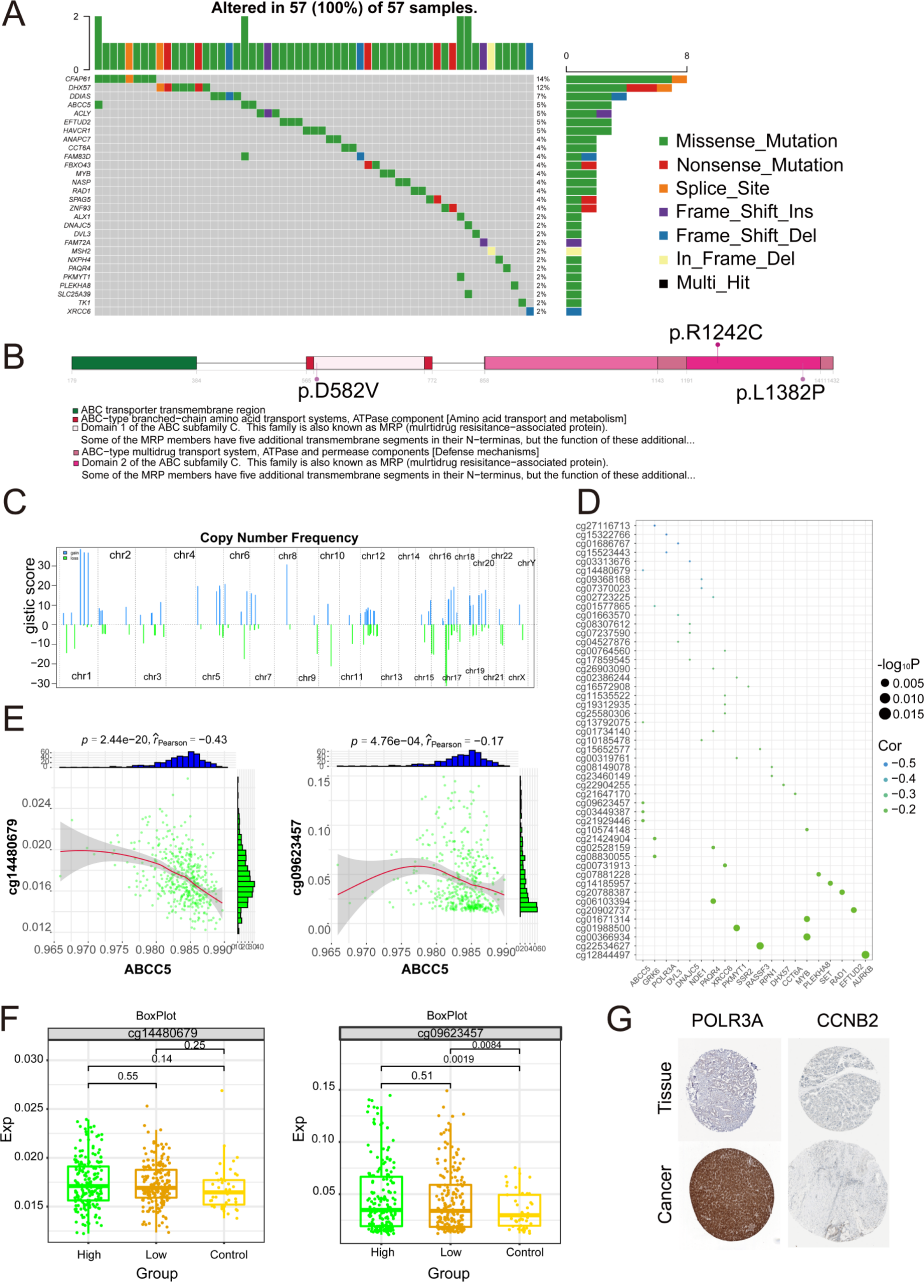
Multi-omics landscape of global regulatory network of ABCC5-based clinical scoring genes. **(A)**. Waterfall map showing the mutant landscape SNPs of ABCC5-based clinical score genes in HCC; **(B)**. The lollipop chart shows the mutation details of the ABCC5-based clinical score gene in HCC; **(C)**. The chromosome table was intended to show the chromosome copy number profile of the ABCC5-based clinical score gene in HCC; **(D)**. Bubble map shows the correlation between ABCC5-based clinical score genes and methylation sites; **(E)**. Scatter plot shows the correlation between the methylation modification level and transcription level of the ABCC5-based clinical score gene global regulatory network in HCC; **(F)**. Box diagram shows the methylation modification level of ABCC5-based clinical score gene global regulatory network in HCC; **(G)**. Immunohistochemical maps showing protein levels of ABCC5-based clinical score genes in HCC.

To further investigate the somatic mutations of ABCC5-based clinical scoring gene in HCC. The results showed that the 28 mutated ABCC5-based clinical score genes had different mutation frequencies (**Figure 9A**), among which the types of mutations were mainly missense mutations, nonsense mutations, and loci mutations. The mutation frequency of ABCC5 was 5%, and the mutation type was missense mutation. A lollipop chart was used to show the mutation site of ABCC5 gene (**Figure 9B**), and the chromosome bar chart showed that the global regulatory network of ABCC5-based clinical score genes in HCC showed copy number increase and deletion (**Figure 9C**), which was particularly obvious in chr1 and chr17. The bubble map shows the methylation regulatory sites of these genes (**Figure 9D**), and correlation analysis shows that the methylation level of ABCC5 and other genes at cg14480679 (*p* < 0.01, r = -0.43) and cg09623457 (*p* < 0.01, r = -0.17) was significantly negatively correlated with the transcription level (**Figure 9E**). The methylation modification level and transcription level of the methylation sites negatively correlated with ABCC5 and other genes in HCC were explored (**Figure 9F**), and the results showed that the low expression of ABCC5 gene may be related to the modification of some proteins by active methylates. Immunohistochemical map showing protein expression of POLR3A and CCNB2 genes in patients with HCC, where POLR3A was highly expressed in patients and CCNB2 was expressed *in vivo* (**Figure 9G**).

In summary, the copy number increase and deletion of ABCC5-based genes in patients with HCC led to missense mutations in the gene loci, resulting in errors in certain genes encoding proteins and contributing to the progression of tumor cells. Meanwhile, correlation analysis revealed that the methylation level of ABCC5-based genes showed a significant negative correlation with the transcription level of the genes, which might indicate that the expression of ABCC5-based genes caused some active methylated substances to affect protein modification.

## 4 Discussion

In recent years, molecular targeted therapies and immunotherapies for HCC have become prominent areas of research. ABCC5, an important drug transporter, plays a pivotal role in the onset, progression, and drug resistance of HCC (45, 46). Studies have highlighted the elevated expression of ABCC5 in liver diseases and its substantial impact on HCC progression, garnering significant attention. High levels of ABCC5 expression are closely associated with patient survival, indicating its potential as a biomarker for HCC (10). ABCC5 may induce drug resistance in tumor cells by regulating drug transport, thus impairing the effectiveness of chemotherapy (12, 47). Furthermore, the overexpression of ABCC5 is strongly linked to the activation of Treg cells, which promotes immune evasion by tumor cells and accelerates tumor progression (15, 48). ABCC5 contributes to tumor cell proliferation, migration, and metastasis by activating related signaling pathways (15). Clinical data indicate that high ABCC5 expression correlates with poor survival outcomes and treatment responses in HCC patients, further supporting its potential as a prognostic biomarker (10, 15). These findings underscore the critical role of ABCC5 in HCC progression.

As a potential biomarker for HCC, ABCC5 demonstrates higher specificity and sensitivity in early diagnosis and prognostic assessment compared to AFP (14, 49, 50). Unlike stem cell markers such as CD133 and GPC3, ABCC5 plays a more significant role in critical processes such as drug resistance and cell proliferation (51, 52). Furthermore, compared to other ABC transporters like ABCC1 and ABCC4, ABCC5 more directly reflects HCC progression and drug resistance, making it highly valuable for precision medicine and targeted therapies in liver cancer. This study analyzed the ABCC5 gene and found significantly elevated expression in HCC patients, with ROC curve analysis showing its outstanding performance in HCC diagnosis. Survival analysis further validated the strong association between high ABCC5 expression and poor prognosis in HCC patients. By combining evidence from the ROC curve, survival analysis, and ABCC5 transcript levels, this study confirms the potential value of ABCC5 as a biomarker for HCC and emphasizes its crucial role in both diagnosis and prognosis of HCC.

We also found that the genes in the ABCC5-associated modules were significantly enriched in biological functions such as RNA splicing and spliceosome-mediated mRNA splicing, which promote HCC proliferation, migration, and chemotherapy resistance through various mechanisms. Specifically, these biological processes may lead to increased cell proliferation, reduced apoptosis, enhanced migration and metastatic potential, elevated chemotherapy resistance, and immune escape. High expression of ABCC5 may help tumor cells evade immune surveillance and drug inhibition by altering metabolic pathways or inducing mutations (53). Extensive studies have shown a close correlation between ABC transporter genes and cancer drug resistance (54, 55). Furthermore, high expression of ABCC5 may lead to RNA splicing abnormalities, further enhancing resistance to chemotherapy drugs (56). When tumor cells are exposed to anticancer drugs, they may avoid drug inhibition or cytotoxic effects by altering metabolic pathways or inducing mutations (14, 57). In this study, the ABCC5 scoring module genes were significantly enriched in cell cycle signaling pathways, which involve various regulatory proteins that play key roles in cancer progression (58). Existing research indicates that in HCC patients receiving nilotinib treatment for 12 months, high expression of ABCC4 and ABCC5 pumps out the drug, downregulating ABCD3, and inducing upregulation of chromosomal replication, cell proliferation, DNA replication, and DNA damage checkpoints (59). Therefore, high expression of ABCC5 exacerbates tumor progression and chemotherapy resistance by regulating RNA splicing, cell cycle signaling pathways, and immune escape mechanisms (60). Consequently, the development of ABCC5 inhibitors as a targeted therapy strategy may help slow down tumor cell proliferation, enhance chemotherapy efficacy (15), restore antitumor immune responses, and correct RNA splicing abnormalities, thereby overcoming chemotherapy resistance and improving patient prognosis (43). However, despite the significant potential of ABCC5 in HCC treatment, current research still has some limitations. First, most studies have focused on the expression levels of ABCC5 in a single disease stage, without comprehensively investigating its role across different stages of HCC development. Second, while ABCC5 plays an important role in drug resistance mechanisms as a drug transporter, its specific molecular mechanisms remain complex and not fully understood. Therefore, future research should further validate the effects and safety of ABCC5 as a target through preclinical models and large-scale clinical cohorts.

Looking ahead, research on ABCC5 should focus on its role in the TME, particularly its impact on immune cell infiltration. Studies suggest that ABCC5 promotes tumor cell immune escape by regulating the activity of immune cells within the TME, especially the proliferation of Treg cells, thereby exacerbating immune resistance in tumors (61). The high expression of ABCC5 in HCC may promote tumor resistance by participating in immune microenvironment reprogramming, particularly through modulating immune cell infiltration. ABCC5 is closely associated with immune escape mechanisms, primarily by regulating the expression of immune checkpoint molecules such as PD-1 and CTLA-4, thereby facilitating immune resistance. Notably, elevated ABCC5 expression shows significant correlation with immune cell abundance, immune checkpoint genes, and tertiary lymphoid structure markers. Targeting ABCC5 and its associated genes could potentiate antitumor immune responses and potentially improve patient outcomes. Moreover, the upregulation of immune checkpoints within TME promotes immune escape, and their blockade may enhance antitumor immunity. In this context, ABCC5-targeted interventions could amplify these immune responses. However, challenges including TME heterogeneity and drug resistance necessitate further investigation. Future research should focus on: elucidating the intricate interplay between ABCC5-mediated immune reprogramming and therapeutic resistance, developing ABCC5-specific inhibitors, and exploring combination immunotherapy strategies. These approaches require rigorous preclinical and clinical validation to evaluate their safety and therapeutic efficacy.

The exploration of upstream regulators of gene sets based on the construction of ABCC5 score identified upstream regulators such as CENPA, ERCC6, among others. CENPA, as a transcriptional regulator, was significantly upregulated in HCC and correlated with poor prognosis of HCC patients (62), which can be used as a potential diagnostic and prognostic marker for HCC. In addition, potential drug targets regulating the ABCC5-based scoring gene set were identified, while a total of 50 docking sites were molecularly docked by molecular docking ABCC5 with drug small molecules. And the drug molecule ZDV can be used as an antiretroviral drug for the treatment of human immunodeficiency virus infection, which alters pyrimidine metabolism and protein homeostasis-related transcription (63, 64). ABCC5 overexpression leads to poor prognosis in patients with HCC. ZDV, a drug primarily used to treat HIV, shows potential as a therapeutic agent for HCC, especially in the context of high ABCC5 expression. ABCC5, a key transporter protein, plays a crucial role in pyrimidine metabolism and protein homeostasis—processes essential for cancer cell survival and proliferation. Research indicates that ABCC5 regulates nucleotide transport, which in turn affects the intracellular concentration of drugs like ZDV (60, 65). ZDV therapeutic potential in HCC is closely related to its ability to modulate ABCC5 expression, influencing cellular responses to nucleoside-based drugs and potentially inhibiting tumor growth.

By integrating clinical scoring and models based on ABCC5, this study provides an in-depth exploration of the role of ABCC5 and its related pathways in disease progression and treatment outcomes. This approach offers new perspectives on the re-utilization of ZDV and other nucleoside analogs in HCC treatment, emphasizing the importance of targeting the transporter protein ABCC5 to improve drug delivery and overcome cancer drug resistance (12). The construction of ABCC5-based clinical scoring and models contributes to the effective exploration of key factors influencing the impact of target genes and their related genes on disease. Overall, this study uncovers potential etiological factors and drug targets in HCC, providing new ideas for treatment research. However, limitations remain, and future research could validate these findings using patient-derived models or larger clinical cohorts, along with single-cell data analysis. Additionally, experimental techniques such as CRISPR-Cas9 could be utilized to further investigate ABCC5 functionality. Further *in vitro* and *in vivo* studies would also help evaluate the potential of ABCC5 inhibitors in overcoming resistance and enhancing treatment outcomes.

This study provides a comprehensive analysis of the multi-omics profile of ABCC5 clinical scoring genes, identifying mutated genes such as CFAP61 and ABCC5, with missense mutations being the most common. Missense mutations result in the abnormal protein function of ABCC5-related genes, altering the distribution and metabolic balance of intracellular drugs (66). Additionally, the stability and activity of proteins may be disrupted, triggering various pathological changes (67). In HCC, missense mutations reveal the role of ABCC5 and its related genes in tumors, driving the development of targeted therapies to regulate the stability of tumor suppressor proteins or optimize existing treatment strategies (68). Research on missense mutations provides a foundation for personalized treatment using chemotherapy drugs and targeted therapies (69). Therefore, a deeper understanding of the functional consequences of missense mutations is crucial for elucidating disease molecular mechanisms and identifying potential therapeutic targets. Currently, there is limited research on the prognosis models of ABCC5-related genes, and future studies should focus on developing novel prognostic models to support clinical decision-making and improve patient prognosis. Validation studies and clinical trials are essential to assess the clinical applicability of these findings. Additionally, this study found that the proliferation of cancer cells in HCC patients is associated with chromosomal replication, DNA replication, and DNA damage, consistent with existing literature (59). The phenomena of insertions and deletions in the chromosomal copy number spectrum are particularly significant on chromosomes 1 and 17. ABCC5 clinical scoring genes are significantly negatively correlated with methylation modifications in HCC. RNA methylation modifications not only affect mRNA but also involve the transcription, splicing, translation, localization, and stability of lncRNAs. In conclusion, the mutation spectrum provided in this study helps explain the poor prognosis of HCC patients and reveals the value of multi-dimensional integrative analysis in cancer biology research, further emphasizing the significance of post-transcriptional modifications in cancer progression, with data supporting both mRNA and lncRNA levels.

The exploration of multi-omics biomarkers provides a comprehensive understanding of disease mechanisms, enhances predictive accuracy, and helps identify targets for early diagnosis, prognosis, and treatment monitoring. This study, through the integration of multi-omics data, is the first to propose ABCC5 as a potential biomarker for HCC and reveals its roles in tumor progression, drug resistance, and immune evasion. The ABCC5 gene scoring model is significantly correlated with patients’ survival rates and pathological staging. It accurately predicts clinical outcomes in both the training and external validation sets (TCGA-LIHC, GSE76427), demonstrating its potential in clinical decision-making. However, the study has limitations. Firstly, the GSE76427 dataset has a relatively small sample size, which may limit the model’s generalizability. A smaller sample size could result in poor applicability across different populations, necessitating an expansion of the sample size and the inclusion of more heterogeneous patient groups. Additionally, the existing datasets may not fully represent the global HCC patient population, as regional, ethnic, and clinical practice differences may affect the universality of the results. Future studies should incorporate samples from diverse geographical and ethnic backgrounds to verify the broad applicability of the ABCC5 model. Despite these challenges, ABCC5 and its related genes likely play a crucial role in liver cancer progression through complex regulatory networks, particularly within the TME, which requires further investigation. Future research should delve deeper into the mechanisms of ABCC5 in immune evasion and immune cell infiltration, and validate its role through larger-scale clinical data. Additionally, the development of ABCC5-targeted inhibitors and the integration of multi-omics studies hold promise for providing a new theoretical foundation for personalized liver cancer treatment.

## 5 Conclusion

This study elucidates the critical role of ABCC5 in HCC. Our findings demonstrate that ABCC5 is significantly overexpressed in HCC tissues and strongly associated with poor patient prognosis, suggesting its potential as a diagnostic and prognostic biomarker for HCC. Multi-omics analyses reveal that ABCC5 participates in regulating key biological processes including cell cycle, RNA splicing, and p53 signaling pathway, thereby promoting HCC progression. Notably, ABCC5 is involved in the reprogramming of HCC immune microenvironment, with its high expression closely correlated to immunosuppressive characteristics such as increased Treg infiltration. Experimental validation confirms that the antiviral drug ZDV exerts antitumor effects by specifically inhibiting ABCC5 and activating the p53 pathway. The ABCC5-based clinical model developed in this study provides a novel tool for prognostic evaluation and personalized treatment of HCC patients. These findings offer important insights into the molecular mechanisms of HCC pathogenesis and facilitate the development of new therapeutic strategies.

## Data Availability

Publicly available datasets were analyzed in this study. This data can be found here: Publicly available datasets were analyzed for this study. RNA-seq data for TCGA_LIHC were obtained from https://portal.gdc.cancer.gov/.

## Supplementary material

Supplementary Figure S1 DCA analysis assesses the ABCC5 model prognosis after including tumor staging.

Supplementary Figure S2 Validating model generalizability.
