# Supplementary material for: To explore the prognostic efficacy and mechanism of ABCC5 clinical scoring model in hepatocellular carcinoma
Source: Front Oncol. 2025 Aug 11;15:1519533. doi: 10.3389/fonc.2025.1519533 (PMC12375932; doi:10.3389/fonc.2025.1519533)

Figure S1: DCA analysis assesses the ABCC5 model prognosis after including tumor staging

The ABCC5 scoring model consistently demonstrates a greater net benefit at various threshold levels compared to models that include tumor staging, highlighting its superior ability to guide clinical decisions. Its decision curve surpasses those of other models, emphasizing its potential to optimize clinical outcomes and reinforcing its value for clinical practice.

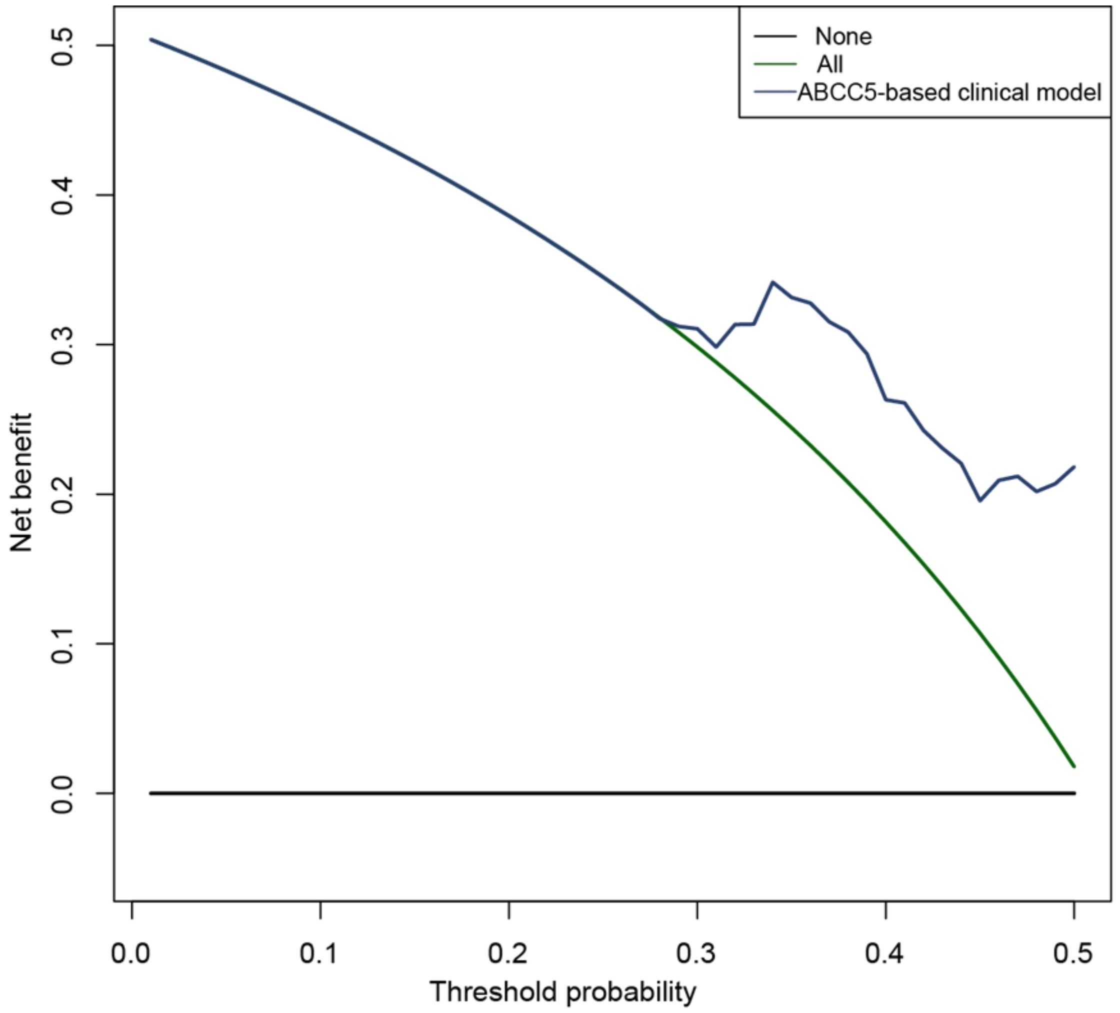

Supplement: Supplementary Figure S1 — DCA analysis assesses the ABCC5 model prognosis after including tumor staging. [file Image1.pdf]
