# Supplementary material for: To explore the prognostic efficacy and mechanism of ABCC5 clinical scoring model in hepatocellular carcinoma
Source: Front Oncol. 2025 Aug 11;15:1519533. doi: 10.3389/fonc.2025.1519533 (PMC12375932; doi:10.3389/fonc.2025.1519533)
Supplement: Supplementary Figure S2 — Validating model generalizability. [file Image2.pdf]

Figure S2: Verify the model's generalizability

To further evaluate the generalizability of the ABCC5-based model, we performed an analysis of RNA-seq data from GSE76427, which includes tumor tissue samples from 115 HCC patients and 52 adjacent normal tissue control samples. Principal component analysis (PCA) scatter plot analysis showed clear differentiation between tumor and normal tissue samples, with good reproducibility within each group, confirming the robustness of the data (Figure S2A). Following this, we conducted validation of the ABCC5 scoring model using the clinical data we had collected, demonstrating its applicability in a clinical context (Figure S2B). Furthermore, survival analysis using Kaplan-Meier curves revealed that the ABCC5-based model was a strong predictor of patient prognosis, further supporting its clinical relevance (Figures S2C-S2D). We compared the performance of existing models using timeROC-TimeDependent, which demonstrated high accuracy in predicting patient prognosis (Figure S2E). These results collectively highlight the potential of the ABCC5 scoring model as a reliable prognostic tool in HCC. The TNM model achieved a peak AUC of 0.76 at 730 days and maintained a stable long-term AUC of 0.73. The BCLC model demonstrated consistent performance in 2–3-year predictions (AUC=0.71) but showed weaker short-term performance (0.58). The ABCC5 model exhibited comparable performance to TNM in short-term (0.61) and intermediate-term predictions (0.75), but displayed significant performance decline in long-term assessments (0.71). The AGE model only showed predictive value within 1 year (0.62), with its performance approaching random chance levels (0.51) in long-term evaluations (Figure S2F).

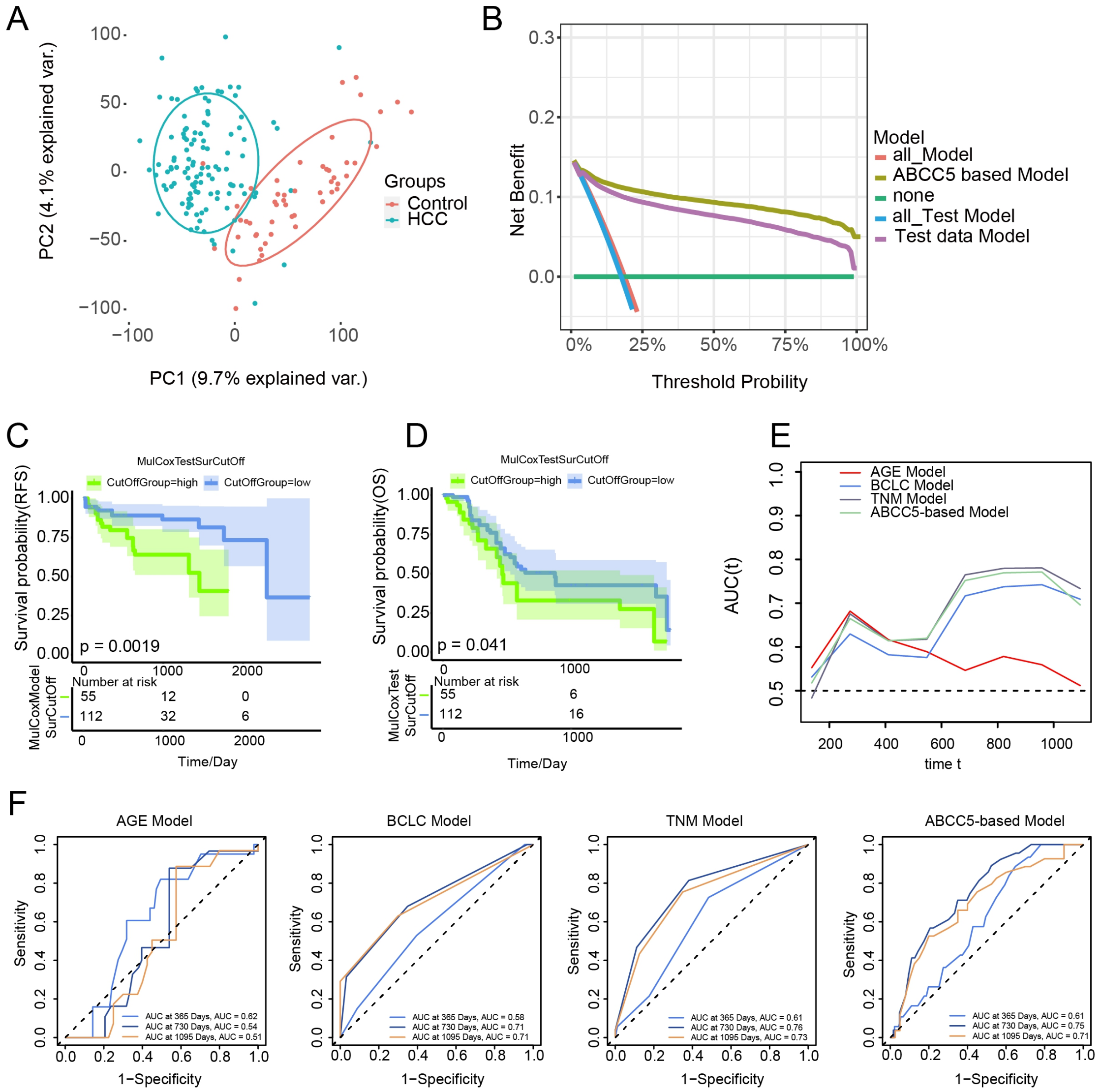

Figure S2: Verify the model's generalizability

(A). PCA density map mapping ABCC5 expression values; (B). Decision Curve Analysis predicts the net benefit of the model-estimated risk probabilities at the risk thresholds; (C). Survival curve Displays the RFS survival curve of the ABCC5-based score; (D). Survival curve Displays the OS survival curve based on the ABCC5-based score; (E). The ROC curves demonstrate the prognostic generalizability of the models; (F). The AUC curves serve as a standard for assessing the performance of predictive models.
